# Interobserver variability and likelihood of malignancy for fifth edition BI-RADS MRI descriptors in non-mass breast lesions

**DOI:** 10.1007/s00330-019-06312-7

**Published:** 2019-08-07

**Authors:** Magdalena Lunkiewicz, Serafino Forte, Bianka Freiwald, Gad Singer, Cornelia Leo, Rahel A. Kubik-Huch

**Affiliations:** 1 Department of Radiology, Kantonsspital Baden, Im Ergel 1, CH-5404 Baden, Switzerland; 2 Medizinisch Radiologisches Institut (MRI) Bahnhofplatz, Bahnhofplatz 3, 8001 Zürich, Switzerland; 3 Department of Pathology, Kantonsspital Baden, Im Ergel 1, CH-5404 Baden, Switzerland; 4 Department of Gynaecology, Kantonsspital Baden, Im Ergel 1, CH-5404 Baden, Switzerland

**Keywords:** Image-guided biopsy, DCIS, Breast, Magnetic resonance imaging, Interobserver variability

## Abstract

**Objective:**

Non-mass enhancement (NME) in breast MRI is the most common feature of ductal carcinoma in situ (DCIS). We sought to evaluate the interobserver variability and positive predictive value (PPV) for malignancy of NME descriptors using the fifth edition BI-RADS lexicon focusing on the newly introduced “clustered ring enhancement” pattern.

**Materials and methods:**

Breast MRIs of 129 patients who had undergone MRI-guided vacuum-assisted biopsy (VAB) in our institution were reviewed. Studies assessed as NME were classified according to the fifth edition BI-RADS lexicon by two breast radiologists. Consensus was reached by involving a third radiologist. Interobserver variability and PPV for malignancy were assessed.

**Results:**

Seventy-two of 129 studies were assessed as NME. The disagreement rate in the first assessment step (mass vs. NME) was low at 9.3% (*ĸ* = 0.81, 95% confidence interval [CI] 0.71–0.91). The disagreement rate for distribution patterns was 23.6% (*ĸ* = 0.67, 95% CI 0.54–0.80) and 22.2% (*ĸ* = 0.69, 95% CI 0.56–0.81) for internal enhancement patterns. Clustered ring enhancement (PPV 53.85, *p =* 0.038) and segmental distribution (PPV 62.5%, *p =* 0.028) had the highest malignancy rates among internal enhancement and distribution patterns with a significant result; the combination of clustered ring enhancement and segmental distribution raised the malignancy rate by approximately 4% (PPV 66.67%, *p* = 0.049).

**Conclusion:**

There was a high agreement rate among readers when differentiating NME from mass lesions. The agreement rate was lower when assessing the distribution and internal enhancement pattern descriptors, but still substantial. The descriptors clustered ring enhancement and segmental distribution were significant predictors of malignancy.

**Key Points:**

*• Non-mass enhancement is a common morphological feature of non-invasive breast cancer (DCIS) in MRI. Differentiation between potentially malignant and benign changes may be very challenging.*

*• Since clustered ring enhancement and segmental distribution are both significant predictors of malignancy, the awareness of this important finding, combined with high-quality image interpretation skills, may improve the tumor detection rate.*

*• The combination of clustered ring enhancement and segmental distribution increases the positive predictive value for malignancy, which may be relevant for clinical practice*.

## Introduction

Magnetic resonance imaging (MRI) has the highest sensitivity (88–100%) among breast imaging modalities [1–3]. Its relatively lower specificity of 72% [1] is considered to be its main disadvantage [4].

In the literature, MRI findings are reported to correlate with second-look ultrasonography examinations in 11–65% [5–7] of cases; this wide range might be attributable to factors such as equipment, experience of the radiologists, patient selection, or type of the lesion (mass, NME, or focus). Lesions that are only detectable on MRI (“MRI-only lesions”) attest to its high sensitivity and to the crucial role of MRI in breast diagnostic [8], especially in high-risk patients.

According to the Breast Imaging Reporting and Data System Lexicon (BI-RADS) [9], non-mass enhancement (NME) represents an area of contrast enhancement without a space-occupying effect. NME is the most common morphologic feature of ductal carcinoma in situ (DCIS). It does not have a sonographic correlate in the majority of cases (NME correlation rate 12% vs. mass lesion correlation rate 65%) [7, 10], which implies the need for MRI-guided biopsy. Evaluation of NME lesions is challenging and it is associated with more false-positive results in comparison with enhancing mass lesions [11–14]. High-quality imaging-based characterization, while subject to interobserver variability, may improve the differentiation between potentially malignant and benign lesions and possibly contribute to the reduction of unnecessary biopsies.

BI-RADS lexicon is the main source of breast imaging terminology, reporting standards, and classification systems for mammography, ultrasound, and MRI of the breast. It provides a uniform assessment structure with recommendations for management. This facilitates intra- and interdisciplinary communication. The first edition of the BI-RADS lexicon was published 1993, followed by four more editions in 1995, 1998, 2003, and the currently used fifth edition in 2013. In the fifth edition of the BI-RADS lexicon [9], a few MRI terms for internal enhancement and distribution patterns of NME have been slightly changed. In the category of distribution patterns, “ductal enhancement” has been eliminated [9, 15]. Also the internal enhancement pattern descriptor “reticular/dendritic” has been omitted, while the descriptor “clustered ring enhancement” has been introduced. Clustered ring enhancement pattern is defined as multiple thin rings of enhancement, made visible around the ducts due to the enhancement of periductal stroma [14]. According to several studies [16–21], clustered ring enhancement is associated with one of the highest malignancy rates (Fig. 3).

The goal of our study was to assess interobserver variability when evaluating breast lesions on MRI according to the fifth edition of the BI-RADS MRI lexicon, as well as to evaluate the positive predictive value (PPV) for malignancy of each NME subtype, focusing on the newly introduced descriptor “clustered ring enhancement.” Awareness of the values in malignancy prediction for NME descriptors, combined with high-quality image interpretation skills, may improve the tumor detection rate.

## Materials and methods

### Patients

This study was approved by the hospital ethics committee (project ID: 2017-00333). Because of the retrospective nature of the data retrieval, specific written consent was waived.

All patients (*n* = 144) who had undergone an MRI-guided vacuum-assisted core breast biopsy (VAB) between January 2011 and May 2017 in our institution were identified from the Picture Archiving and Communications System (PACS).

The inclusion criterion was the presence of the histopathological diagnosis based on the MRI-guided biopsy.

### Breast MRI examination, MRI-guided VAB, and histopathological diagnosis

Diagnostic MRI examinations in our institution were performed in the prone position, using a 1.5-T scanner (Magnetom Aera®, Siemens Healthcare) in 89 cases and a 3-T scanner (Skyra®, Siemens) in 11 cases, as it had just become available at our new site. All examinations have been performed with a dedicated breast coil (18 channels). From the total number of 144 initially identified MRI examinations prompting an MRI-guided biopsy, 34 cases have been performed in external institutions (Siemens Avanto®, Area®, Espree®, Verio®; Philips Healthcare Ingenia®, Philips Inc.). Of the 34 external MRI examinations, 19 cases were performed on a 1.5-T scanner, 10 cases were performed on a 3-T scanner, and 5 examinations were excluded because of incomplete data sets, resulting in a total of 129 data sets for analysis. This resulted in a total number of 108 cases performed on a 1.5-T and 21 cases performed on a 3-T scanner.

The imaging protocol for the 1.5-T system included pre-contrast turbo spin-echo, T2-weighted axial images (T2WI) (repetition time TR 5600 ms; echo time TE 110 ms; FOV 360 mm; matrix 512; section thickness 3.0 mm), one pre-contrast dynamic T1-weighted axial (T1WI), and five post-contrast dynamic fat-saturated axial T1WI (repetition time TR 4.87 ms; echo time TE 2.39 ms; FOV 360 mm; matrix 448; section thickness 1.0 mm, flip angle 10°). The post-contrast series images were acquired 1 min 45 s, 3 min 14 s, 4 min 44 s, 6 min 14 s, and 8 min 30 s after the intravenous injection of Gd-DOTA (Dotarem®, Guerbet) (0.1 mL/kg body weight).

In the post-processing step, image subtractions were performed for each phase in the axial plane. In the sagittal and coronal planes, the subtractions were obtained in the early phase. Motion artifacts were corrected using commercial software (3 D Elastic Motion Correction®, Siemens Medical Solutions).

The MRI-guided VAB technique, as previously described by our group [8, 22], was performed according to the Swiss Minimally Invasive Breast Biopsies Working Group (MIBB) guidelines [23], with an 8-gauge needle [24].

### Image interpretation

**Fig. 1:**
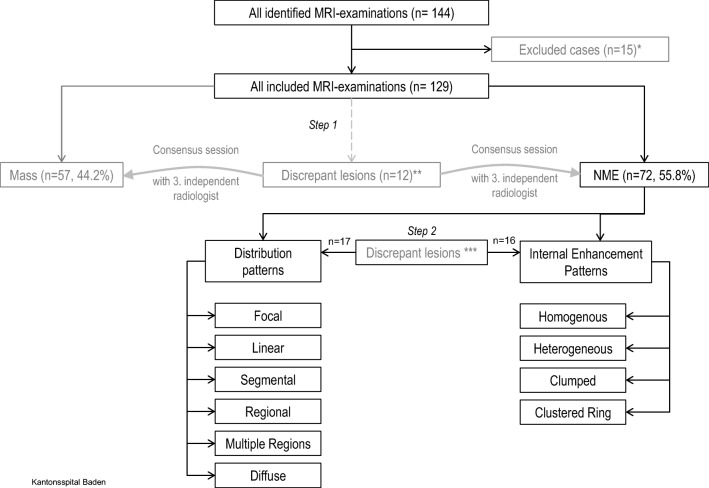
Flowchart of the overall design, which involves the key steps of the prospective lesion evaluation, according to the revised BI-RADS lexicon (fifth edition). Step 1: General assessment (mass or NME). Step 2: Evaluation of the internal enhancement and distribution patterns. *Incomplete data set; **12/129, disagreement rate 9.3%, kappa 0.81 (CI 0.71–0.91); ***Discrepant cases in distribution pattern 17/72, disagreement rate 23.6%, kappa 0.67 (CI 0.54–0.80). Discrepant cases in internal enhancement pattern 16/72, disagreement rate 22.2%, kappa 0.68 (CI 0.55–0.81)

**Fig. 2:**
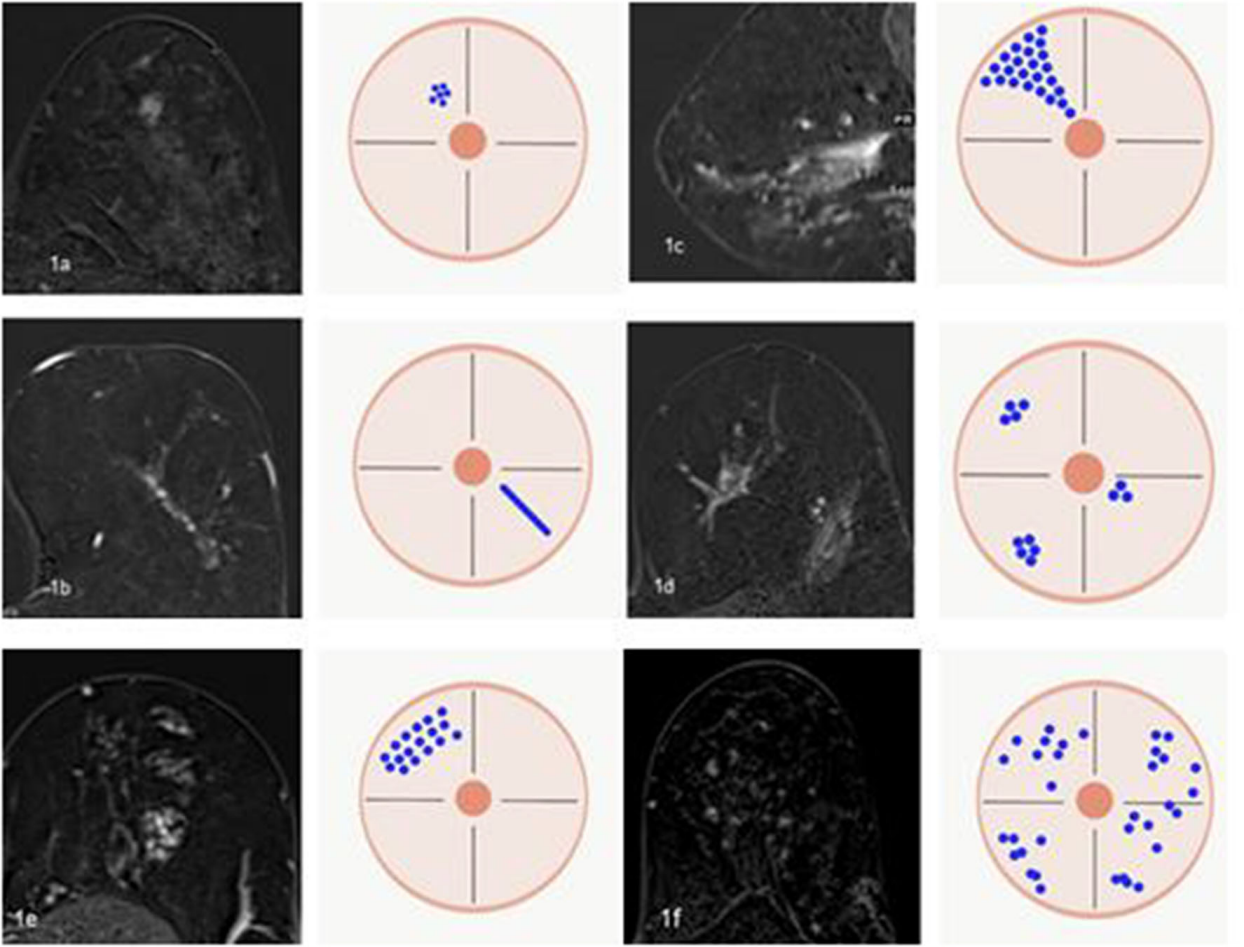
Example cases from our daily practice (Department of Radiology, Kantonsspital Baden, Switzerland) for enhancement distribution patterns according to the fifth edition of BI-RADS lexicon [9]. **1a** Focal (< 25% of quadrant, fat or normal glandular tissue interspersed among the enhancing components): A 49-year-old woman with usual ductal hyperplasia and fibrosis. **1b** Linear (enhancement in a line, may be branching): A 75-year-old woman with linear clumped enhancement pattern and proven ductal carcinoma in situ. **1c** Segmental (triangular, apex pointing to nipple, suggests ductal enhancement): A 60-year-old woman with invasive lobular carcinoma. **1d** Multiple regions (enhancement over at least two large volumes of tissue): A 74-year-old woman with infiltrations of invasive lobular carcinoma. **1e** Regional (encompasses more than a single duct system): A 52-year-old woman with normal parenchyma (dense breasts). **1f** Diffuse (distributed randomly throughout the breast): A 47-year-old woman with fibrocystic changes

**Fig. 3:**
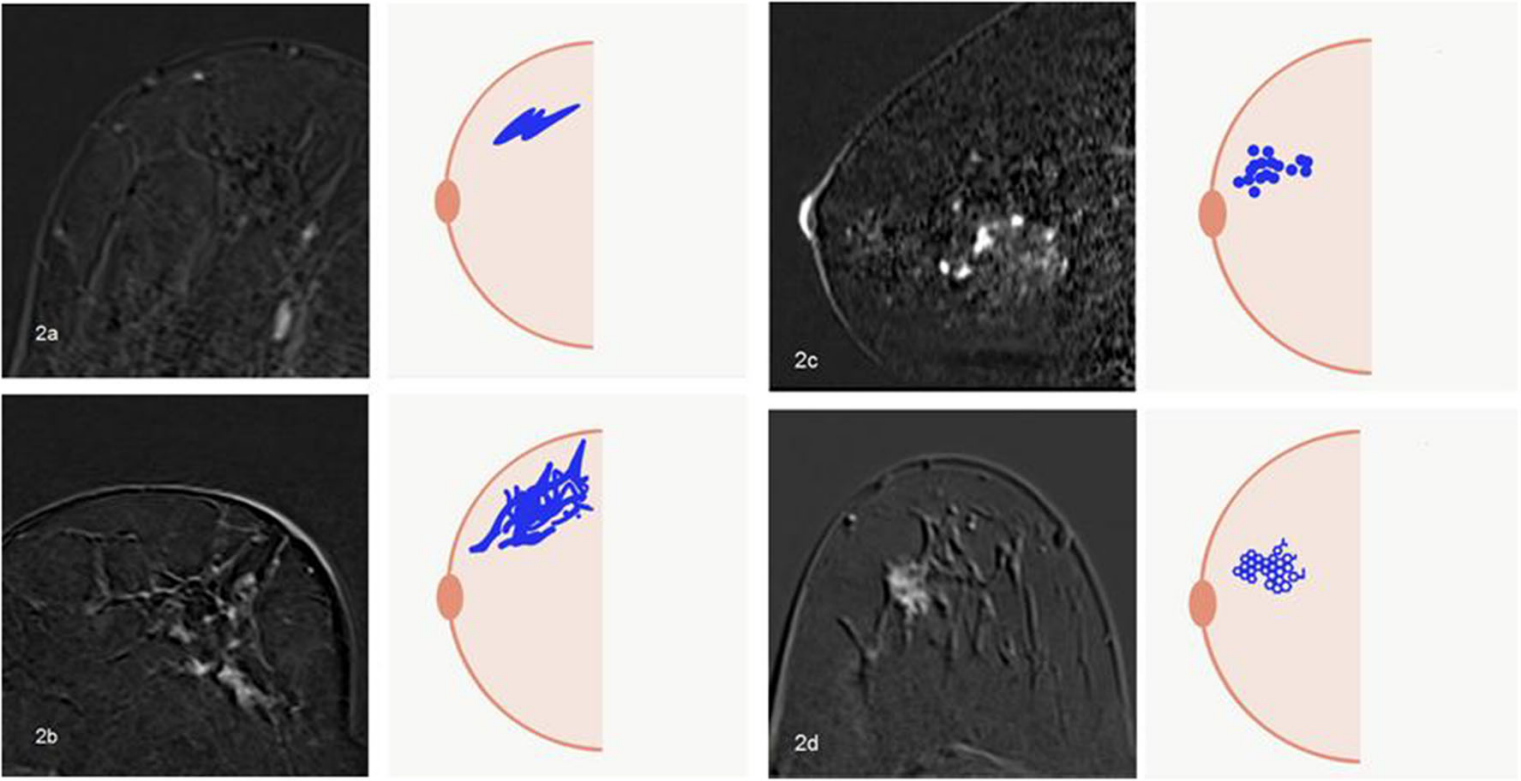
Example cases from our daily practice (Department of Radiology, Kantonsspital Baden, Switzerland) for internal enhancement patterns according to the 5th edition of BI-RADS lexicon [9]. **2a** Homogeneous: A 55-year-old woman with usual ductal hyperplasia. **2b** Heterogeneous: A 56-year-old woman with apocrine focal metaplasia. **2c** Clumped: A 67-year-old woman with pseudoangiomatous stromal hyperplasia and fibrosis. **2d** Clustered ring enhancement: A 65-year-old woman with invasive lobular carcinoma

Two experienced breast radiologists (R.K. and S.F., 20 and 10 years of experience, respectively) independently evaluated all cases de novo using the fifth edition BI-RADS MRI lexicon. Both readers were unaware of the patients’ clinical details, initial radiological reports, and the pathological results. In case of discrepancy, the findings were reviewed together with a third radiologist to achieve consensus. According to the ACR MRI lexicon, lesions were first classified as NME [9] or mass lesions (Fig. 1). These findings were visually assessed, based on the morphologic characteristics in the turbo spin-echo T2WI sequence in the axial plane (presence/absence of mass effect) and post-contrast dynamic T1WI sequences (presence/absence of interspersed normal parenchyma or fat tissue in the enhancing area). All MRI examinations assessed as NME were included for further analysis and subsequently evaluated in the post-contrast dynamic (delay, 1.45 min) and subtraction images (axial, coronal, and sagittal planes) according to the enhancement distribution modifiers (Figs. 1 and 2) and the pattern of internal enhancement (Figs. 1 and 3).

In case of a clustered ring enhancement pattern, the absence of micro-cystic changes, which would indicate benignity, was confirmed in a T2-weighted sequence.

The BI-RADS classification from the original report had been recorded, since both readers were biased knowing that a biopsy had been performed.

### Statistical analysis

With histopathological diagnosis as the reference standard, PPVs of the internal enhancement and global distribution patterns for determining malignancy were calculated as the proportion of malignant lesions to the corresponding pattern.

Any histopathological result other than DCIS or invasive carcinoma was considered benign; B3 lesions (“lesions of unknown biological potential”) [25], which had not undergone subsequent surgery, were excluded from our statistical analysis.

PPV was calculated for each descriptor of internal enhancement and distribution patterns and for their different combinations.

To compare the PPVs of different enhancement patterns, odds ratios (OR) and Fisher’s exact tests were calculated as follows: PPV of the corresponding pattern (malignant/non-malignant) vs. PPV of the other patterns.

To evaluate interobserver agreement, the number of discrepant assessments (reader 1 vs. reader 2) was determined and Cohen’s kappa (ĸ) was calculated. The agreement rate was calculated for both steps (NME vs. mass lesion followed by evaluation of both distribution and internal enhancement patterns) of prospective lesion evaluation (Fig. 1) as well as separately for internal enhancement and distribution pattern assessment*.* Cohen’s kappa values were measured according to the method of Landis and Koch [26].

Descriptive statistics are presented as counts and frequencies for categorical data and mean (standard deviation) for metric variables.

A *p* value < 0.05 was considered significant.

Statistical analysis was performed by using open-source statistical software (R version 3.1.1) [27].

## Results

### Demographics of the study population

The patients’ ages in the study population ranged from 31 to 91 with a mean age of 57.1 years (± 11.6).

From the total number of included studies (*n* = 129), 72/129 (55.8%) were classified as NME. The patients’ ages in the NME group ranged from 31.5–91 with a mean age of 52.6 years (± 20.8).

Of the 72 cases, 18/72 (25%) had a positive family history of breast cancer (as declared by the patient on the MIBB-questionnaire; lifetime risk was not recorded), 17/72 (24%) had no family history, and 37/72 (51%) were not specified.

The study group identified to access the PPV for malignancy (after exclusion of the B3 lesions, which had not undergone surgery) included the total number of 67 cases. The patients’ age in this group ranged from 31.5–81 with a mean age of 51.5 years (± 20.7).

Of the 67 cases, 17/67 (25.4%) had a positive family history of breast cancer (as declared by the patient on the MIBB-questionnaire; lifetime risk was not recorded), 15/67 (22.4%) had no family history, and 35/67 (52.2%) were not specified.

In the overall study population group, 60% (78/129) of lesions had been classified as BI-RADS 4, 24% (31/129) as BI-RADS 3, 3% (4/129) as BI-RADS 5, and 13% (16/129) had unrecorded BI-RADS classifications (mostly images from external sites).

### Interobserver agreement

**Fig. 4:**
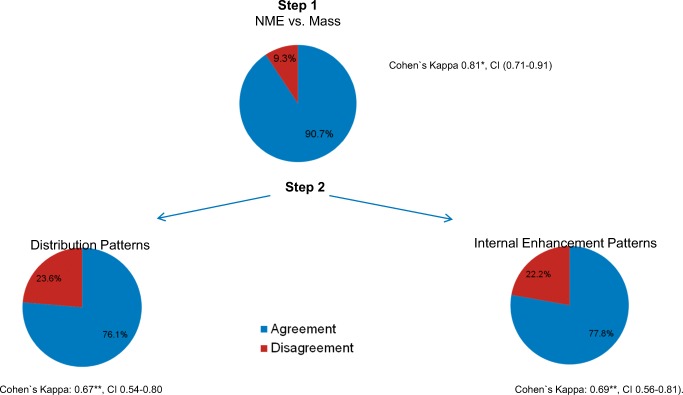
Interreader agreement. *0–0.20, slight agreement; 0.21–0.40, moderate agreement; 0.61–0.80, substantial agreement; and 0.81–1, almost perfect agreement

The interobserver agreement rates for each of the assessment steps are shown in Figs. 1 and 4.

The interobserver agreement rate in the second assessment step (evaluation of internal enhancement and distribution patterns) was lower than in the first assessment step (NME vs. mass lesion), but still substantial for evaluation of both distribution and internal enhancement patterns (Fig. 4).

### Overlap of clustered ring enhancement and other internal enhancement patterns in the visual assessment process (interobserver agreement on clustered ring enhancement)

In 18 cases, one or both readers assessed a study as clustered ring enhancement. In 8/18 cases, the visual assessment between both readers was discrepant (44% disagreement rate). The total number of cases with a definite assessment as clustered ring enhancement after the consensus session with a third independent radiologist was *n* = 14. In 10/14 studies, the visual assessment between both readers was concordant (71% agreement rate).

Of the four remaining discrepant studies, 3 studies were assessed in consensus as a clumped enhancement, and the other 3 were assessed as heterogeneous. There were no further intersections between clustered ring enhancement and other internal enhancement patterns (Table 1).

**Table 1:** Intersections between clustered ring enhancement and other internal enhancement patterns

	Reader 1				
Reader 2	Clumped	Clustered ring	Heterogeneous	Homogeneous	Rim enhancement (mass)
Clumped		3			
Clustered ring	1	10	0	0	1
Heterogeneous		2			
Homogeneous		0			
Rim enhancement (mass)		1			

### Distribution of internal enhancement and distribution patterns in the NME group

**Fig. 5:**
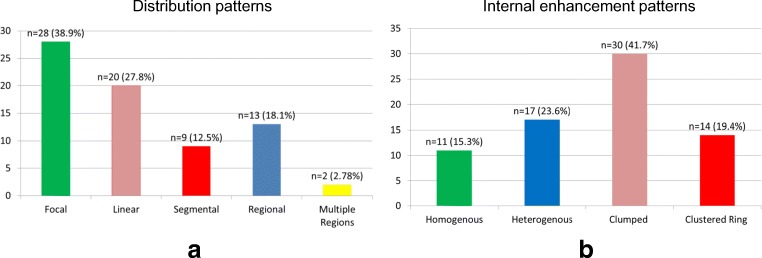
**a**, **b** Distribution of distribution pattern and internal enhancement pattern descriptors in the study population

In the overall NME group, the most frequent distribution pattern was focal (*n* = 28/72, 38.9%), followed by linear (*n* = 20/72, 27.8%), regional (*n* = 13/72, 18.1%), segmental (*n* = 9/72, 12.5%), and multiple regions (*n* = 2/72, 2.78%) (Fig. 5a).

The most frequent internal enhancement pattern was clumped (*n* = 30/72, 41.7%), followed by heterogeneous (*n* = 17/72, 23.6%), clustered ring (*n* = 14/72, 19.4%), and homogenous (*n* = 11/72, 15.3%) (Fig. 5b).

The distribution of internal enhancement and distribution patterns in the study group identified in particular to access the PPV for malignancy correspond to the described above distribution in the overall NME group. The most frequent distribution pattern was focal (*n* = 28/67, 41.8%), followed by linear (*n* = 18/67, 26.9%), regional (*n* = 11/67, 16.4%), segmental (*n* = 8/67, 11.9%), and multiple regions (*n* = 2/67, 3%).

The most frequent internal enhancement pattern was clumped (*n* = 27/67, 40.3%), followed by heterogeneous (*n* = 17/67, 25.4%), clustered ring (*n* = 13/67, 19.4%), and homogenous (*n* = 10/67, 14.9%).

### Histopathological findings in the NME group

The frequency of malignant findings in the overall NME group in our study population was 24.6% (19/72 cases).

**Fig. 6:**
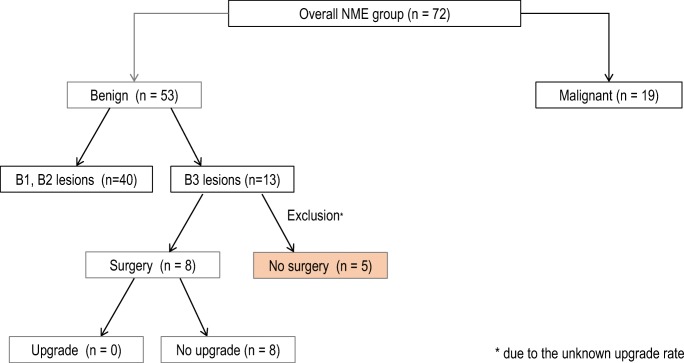
Histopathological diagnoses in the NME group

Of the 53 (73.6%) benign findings, 13 lesions were B3 lesions (18.1% of the total study population). Eight patients with a B3 lesion had subsequently undergone surgical resection and no upstaging had been reported (Fig. 6). The five remaining B3 lesions, which had not undergone subsequent surgery, were excluded from our statistical analysis accessing the PPV for malignancy, due to the unknown upstage rate. The frequency of malignant findings in the consecutive study group was 28.4% (19/67 cases). The distribution of both malignant and benign histopathological diagnoses in both study groups (before and after the exclusion of the selected B3 lesions) is shown in Table 2.

**Table 2:** Distribution of histopathological diagnosis in the overall NME group and NME group*

Overall NME group (*n* = 72)				NME group* (*n* = 67)			
Benign	53 (73.6%)	Malignant	19 (26.4%)	Benign	48 (71.6%)	Malignant	19 (28.4%)
Fibrosis	10 (13.8%)	DCIS	6 (8.3%)	Fibrosis	10 (14.9%)	DCIS	6 (9%)
UDH	6 (8.3%)	IDC	5 (6.9%)	UDH	6 (9%)	IDC	5 (7.5)
ADH	5 (6.9%)	ILC	3 (4.1%)	ADH	3 (4.5%)	ILC	3 (4.5%)
Papilloma Ø atypia	5 (6.9%)	IDC with DCIS	1 (1.7)	Papilloma Ø atypia	4 (6%)	IDC with DCIS	1 (1.5%)
Periductal mastitis	5 (6.9%)	Other malignancy	4 (5.5%)	Periductal mastitis	5 (7.5%)	Periductal mastitis	4 (6%)
Fibroadenoma	4 (5.5%)			Fibroadenoma	4 (6%)		
Sclerosing adenosis	4 (5.5%)			Sclerosing adenosis	4 (6%)		
FEA	2 (2.7%)			FEA	2 (3%)		
Papilloma with atypia	2 (2.7%)			Papilloma with atypia	1 (1.5%)		
Normal parenchyma	1 (1.7%)			Normal parenchyma	1 (1.5%)		
Other benign results	9 (12.5)			Other benign results	8 (11.9%)		

*UDH* usual ductal hyperplasia, *ADH* atypical ductal hyperplasia, *FEA* flat epithelial atypia, *DCIS* ductal carcinoma in situ, *IDC* invasive ductal carcinoma, *ILC* invasive lobular carcinoma

*Consecutive study group after exclusion of the B3 lesions without surgery

### Positive predictive value for malignancy

Of the internal enhancement patterns, clustered ring enhancement had the highest positive predictive value (PPV) for malignancy, significantly higher in comparison with other internal enhancement patterns (53.85%, 7 malignant lesions of a total number of 13 cases, *p* = 0.038). Clumped enhancement, heterogeneous enhancement, and homogenous enhancement were not significant predictors of malignancy (Table 3).

**Table 3:** Positive predictive value for malignancy of distribution patterns and internal enhancement patterns

	Total (*n*)	Malignant	PPV	*p* value	OR
Clustered ring	13	7	53.85	0.038*	3.982
Clumped	27	7	25.93	0.788	0.819
Heterogeneous	17	3	17.65	0.356	0.460
Homogeneous	10	2	20	0.712	0.593
Focal	28	5	17.86	0.177	0.440
Linear	18	5	27.78	1	1.064
Segmental	8	5	62.5	0.028*	5.702
Regional	11	2	18.18	0.714	0.559
Multiple regions	2	1	50	0.468	2.772

Odds Ratios and *p* values from corresponding Fisher’s exact tests were calculated based on the comparison between the PPV of the corresponding pattern (malignant/non-malignant) and other patterns

*p < 0.05 was considered significant

Among the distribution patterns, segmental distribution had the highest PPV for malignancy, which was significantly higher in comparison with other distribution patterns (62.5% %, 5 malignant lesions of a total number of 8 cases, *p* = 0.028). Linear, focal, and regional enhancements were not significant predictors.

The combination of clustered ring enhancement and segmental distribution increased the PPV by approximately 4% to 66.67% (4 malignant lesions of a total number of 6, *p* = 0.049) and represented the highest PPV for malignancy among all possible combinations of internal enhancement and distribution patterns, with a marginally significant result.

## Discussion

As assessment of NME is very challenging, being aware of the interobserver variabilities and their characteristics may improve the quality of reporting. To our knowledge, our study is the first one assessing the interobserver agreement after the publication of the fifth edition BI-RADS lexicon. The interobserver agreement of our study varied strongly between the first assessment step (NME vs. mass lesions) and the second assessment step (differentiation of NME descriptors). The interobserver agreement of the first named assessment step was almost perfect; however, the experience of both readers (20 and 10 years of experience in breast imaging) should be taken into account. The interobserver agreement in the assessment of NME internal enhancement and distribution patterns was considerably lower (by around 14%). Especially in the evaluation of the distribution and internal enhancement patterns, a second reader opinion may be useful. There were overlaps between clustered ring, clumped, and heterogeneous internal enhancement patterns, whose assessment should be perceived as very challenging, especially when performed by less experienced readers.

Apart from the interobserver variability, the results showed that clustered ring enhancement and segmental distribution pattern are significantly more frequently associated with malignancy in comparison with other NME descriptors. This finding is concordant with several previous studies [17, 21, 28, 29]. There are prior studies both with higher and lower absolute results, but there is only one prior study [17] performed after the publication of the revised fifth edition of BI-RADS lexicon (Table 4). This study, by Chikarmane et al, departs from our study in a few aspects. The PPV of clustered ring enhancement for malignancy in our study was 53.85%, which was slightly higher than that observed by Chikarmane et al (44%). This may be related to a different patient selection process; e.g., clustered ring enhancement studies with micro-cystic changes in T2WI sequences were excluded from biopsy, since the coexistence of clustered ring enhancement and micro-cystic changes may be associated with a benign result (mastopathy) [30]. This improved the specificity of our result. Another difference in our study, compared with that of Chikarmane et al, which used two readers, is the achievement of consensus via a third, independent radiologist.

**Table 4:** Tabulated listing of the literature results on clustered ring enhancement and segmental distribution in comparison with our findings

Study	Total no. of included NME lesions	No. of clustered ring enhancement lesions/total (%)	No. of malignant cases/total no. of clustered ring enhancement lesions (PPV %)	No. of lesions with segmental distribution/total (%)	No. of malignant lesions/total no. of lesions with segmental distribution (PPV%)
Tozaki et al [21]	61	23/61 (37%)	22/23 (96%)	19/61 (31%)	19/19 (100%)
Uematsu et al [29]	124	66/124 (53%)	51/66 (77%)	53/124 (43%)	45/53 (85%)
Chikarmane et al [17]*	144	30/144 (21%)	11/30 (37%)	25/144 (17%)	10/25 (40%)
Our study*	67	13/67 (19.4%)	7/13 (53.85%)	8/67 (11.9%)	5/8 (62.5%)

*Performed after the introduction of the revised BI-RADS lexicon 5th edition (2014)

Tozaki et al [21] and Uematsu et al [29] documented a significantly higher malignancy rate and PPV of clustered ring enhancement (96% and 77%, respectively) (Table 4). This may be associated with a different visual assessment concept in those studies, since they perceived the clustered ring internal enhancement descriptor mainly as a subtype/supplement to the heterogeneous and clumped enhancement pattern, as their studies were performed before the publication of the 5th edition of the BI-RADS lexicon 2014. The higher frequency rates of clustered ring enhancement, documented by those two prior studies (Tozaki et al, 23/61–38%; Uematsu et al, 66/124–53%; our study, 13/67–19.4%) and the different methods of confirmation of the histopathological diagnosis between the trials (our study, MRI-guided VAB in 100% of cases; Tozaki et al, additional mastectomy and lumpectomy in 60% of cases) may have influenced the final results.

Among the distribution modifiers, segmental distribution pattern had a significantly higher PPV for malignancy with 62.5% (*p* = 0.028). This finding is consistent with previously published results [17, 21, 28, 29] (Table 4). The observed subtle decrease of the PPV of segmental distribution in comparison with Tozaki et al and Uematsu et al may be again related to our institution’s different indications for MRI-guided VAB (MRI-only lesions). This suggests less distinct lesions in our study group (no sonographic correlate) and correspondingly lower malignancy rates, compared with the studies of Tozaki et al and Uematsu et al (malignancy rate Tozaki et al, 35/61 [57%]; Uematsu et al, 85/124 [69%]; our study, 19/67 [28.4%]).

Our study has several limitations. This was a retrospective study.

The highest PPV for malignancy (66.67%) was assessed for the combination of clustered ring enhancement and segmental distribution. However, this result has almost missed the significance level with a *p* value of 0.049, due to the relatively low number of cases.

The family history of breast cancer was recorded as declared by the patient on the MIBB questionnaire; lifetime risk was not calculated.

Since the included lesions were MRI-only lesions, which tended to be less distinct and subject to higher interobserver variability, we did not evaluate the lesion size; thus, we could not stratify for size. The potential overlap between larger NME lesions and background parenchymal enhancement (BPE) should be also taken into account.

In conclusion, the awareness of higher interobserver variability in the assessment of the distribution and internal enhancement patterns of NME lesions, as well as the awareness of the most frequent intersections between descriptors, is relevant for high-quality reporting and implies the requirement of a second (and possibly a third) reader opinion.

The newly introduced clustered ring enhancement and segmental distribution pattern are significant predictors of malignancy among the NME descriptors. The awareness of this important finding combined with high-quality image interpretation skills may improve the tumor detection rate. The combination of both patterns increases the PPV for malignancy rate by an extra 4% (PPV of the combination of both, 66.7%), which may be relevant for clinical practice.

## Abbreviations

ACR: American College of Radiology
B3: Lesions of unknown biological potential
BPE: Background parenchymal enhancement
DCIS: Ductal carcinoma in situ
FOV: Field of view
MIBB: Minimally Invasive Breast Biopsies Working Group
NME: Non-mass enhancement
OR: Odds ratio
PACS: Picture archiving and communication system
TE: Echo time
TR: Repetition time
VAB: Vacuum-assisted breast biopsy
